# *Trigonella foenum-graceum* (Seed) Extract Interferes with Quorum Sensing Regulated Traits and Biofilm Formation in the Strains of *Pseudomonas aeruginosa* and *Aeromonas hydrophila*

**DOI:** 10.1155/2015/879540

**Published:** 2015-04-27

**Authors:** Fohad Mabood Husain, Iqbal Ahmad, Mohd Shahnawaz Khan, Nasser Abdulatif Al-Shabib

**Affiliations:** ^1^Department of Agricultural Microbiology, Aligarh Muslim University, Aligarh 202002, Uttar Pradesh, India; ^2^Department of Food Science and Nutrition, College of Food and Agricultural Sciences, King Saud University, Riyadh 11451, Saudi Arabia; ^3^Department of Biochemistry, Protein Research Chair, College of Sciences, King Saud University, Riyadh 11451, Saudi Arabia

## Abstract

*Trigonella foenum-graecum* L. (Fenugreek) is an important plant of the Leguminosae family known to have medicinal properties. However, fraction based antiquorum sensing and antibiofilm activities have not been reported from this plant. In the present study *T. foenum-graecum* seed extract was sequentially fractionated and sub-MICs were tested for above activities. The methanol fraction of the extract demonstrated significant inhibition of AHL regulated virulence factors: protease, LasB elastase, pyocyanin production, chitinase, EPS, and swarming motility in *Pseudomonas aeruginosa* PAO1 and PAF79. Further, QS dependent virulence factor in the aquatic pathogen *Aeromonas hydrophila* WAF38 was also reduced. Application of *T. foenum-graecum* seed extract to PAO1, PAF79, and WAF38 decreased the biofilm forming abilities of the pathogens by significant levels. The extract also exhibited reduced AHL levels and subsequent downregulation of *lasB* gene. *In vivo* study showed an enhanced survival of PAO1-preinfected *C. elegans* after treatment with extract at 1 mg/mL. Further, the major compound detected by GC-MS, caffeine, reduced the production of QS regulated virulence factors and biofilm at 200 *µ*g/mL concentration indicating its role in the activity of the methanol extract. The results of the present study reveal the potential anti-QS and antibiofilm property of *T. foenum-graceum* extract and caffeine.

## 1. Introduction

Formation of biofilm by many pathogens is closely associated with density dependent cell-cell communication known as quorum sensing (QS), in which small diffusible signaling molecules called autoinducers regulate gene expression. Quorum sensing helps bacterial populations to switch from acting as individual cells to operating in a concerted, multicellular fashion [1]. In clinical settings, biofilms are major threat and challenge because bacteria living within the mode are more protected against host immune responses and are significantly more resistant to various antimicrobial drugs [2, 3].* Pseudomonas aeruginosa* is an opportunistic, nosocomial, and biofilm forming gram negative pathogen that has three main QS pathways. The rhlI/rhlR and lasI/lasR pathways are (acyl homoserine lactone) AHL based and PQS-MvfR pathway is regulated by 2-heptyl-3-hydroxy-4(1 H)-quinolone signal molecule [4–6].* P. aeruginosa* utilizes these signal molecules for the production of biofilms and virulence factors during pathogenesis. Several studies have also shown that QS deficient* P. aeruginosa* has reduced biofilm forming abilities [7, 8]. The above-mentioned observations imply that the quorum sensing inhibitors (QSIs) may have the potential to circumvent the challenge of combating multidrug resistance in bacteria [6]. Thus, it is envisaged that QS inhibitors will also be equally effective against biofilms formed by pathogenic bacteria.

Plant-derived compounds have been used to treat microbial infections for centuries and are supposed to be safe for human consumption [9]. Screening of plant-derived compounds with improved strategy may facilitate the discovery of compounds that attenuate bacterial pathogenesis/biofilms. It is expected that antipathogenic drugs will generate less pressure for the evolution of resistance as compared to antibiotic therapy [10]. Plant-derived compounds such as ursolic acid, naringenin, cinnamaldehyde, salicylic acid, methyl eugenol, essential oils, and extracts from Indian medicinal plants, garlic, and edible fruits have shown various extents of antibiofilm and quorum sensing inhibitory properties against several pathogens [10, 11]. However the majority of Indian medicinal plants are yet to be screened and evaluated for such novel activities. In our previous study, interference in QS mediated violacein production by crude extracts of* T. foenum-graceum *in CV12472 was observed [12]. Therefore, the present study was undertaken to investigate its anti-QS and anti-biofilm potential in search of effective antipathogenic drug principles against bacterial infections.

*Trigonella foenum-graecum* L. (fenugreek) is an important annual medicinal plant of the Leguminosae family and its leaves and seeds have been used in various illnesses and as a health tonic for a very long time. Fenugreek is known to have hypoglycemic, hypocholesterolaemic, antioxidant potency, digestive stimulant action, and hepatoprotective effects [16]. Recent research demonstrated that fenugreek is a valuable medicinal plant of multipurpose uses and may be used for preparing various products such as steroidal hormones [17]. To the best of our knowledge, there is no report available on the antibiofilm activity of* T. foenum-graceum* against PAO1. Therefore, we have selected this plant and elucidated the broad spectrum anti-QS and antibiofilm activity of methanolic extract against pathogenic bacteria.

## 2. Methods

### 2.1. Bacterial Strain and Growth Conditions

The strains used in this study are listed in Table 1. Unless otherwise stated, all of the strains were grown in LB medium.

### 2.2. Plant Material and Preparation of Extracts

*T. foenum-graceum* (L.) (Fenugreek) was purchased from a local market in Aligarh, India. The voucher specimen (MBD-34/09) was deposited in the Department of Agricultural Microbiology, Aligarh Muslim University, Aligarh, India. Plant extract was prepared as described earlier [18]. Briefly, five hundred (500) grams of dry seed powder was soaked in 2.5 L of methanol for 5 days with intermittent shaking and was filtered through Whatman filter paper number 1 (Whatman Ltd., England). The filtered extract was concentrated to dryness under reduced pressure in a rotary evaporator at 40°C and stored at 4°C for future use.

### 2.3. Determination of Minimum Inhibitory Concentration (MIC)

Minimum inhibitory concentration (MIC) of plant extract against test strains was determined by the microbroth dilution method, using specific dye (p-iodonitrotetrazolium violet) as an indicator of growth as described by Eloff [19]. MIC is defined as the minimum concentration of plant extracts which inhibited the visible growth of test strains.

### 2.4. Effect of Methanolic Extract on Quorum Sensing Regulated Virulence Factors

Effect of sub-MICs of plant extract on virulence factors of* P. aeruginosa* and* A. hydrophila* such as LasB elastase, protease, pyocyanin, chitinase, swarming motility, EPS extraction, and quantification was assessed as described previously [20].

### 2.5. Effect on Biofilm Formation

The effect of* T. foenum-graceum* (seed) extract on biofilm formation was measured using the polyvinyl chloride biofilm formation assay [21]. Briefly, overnight (treated and untreated) cultures of PAO1, PAF79, and WAF38 were visualized for biofilm formation by staining with 0.1% crystal violet solution and measuring the absorbance at OD_470_.

### 2.6. Scanning Electron Microscopy

Biofilms were grown on glass coverslips, in the treated and untreated cultures of PAO1. After 24 h of incubation, the cover slips were rinsed with distilled water to remove planktonic cells and processed for scanning electron microscopy (SEM) examination [20].

### 2.7. Analysis of lasB Transcriptional Activity in* E. coli*

Measurement of *β*-galactosidase luminescence in* E. coli* MG4/pKDT17 was done by the method described previously [20]. Briefly, culture supernatant was extracted by ethyl acetate for quorum sensing signal molecules (AHLs) from overnight cultures of PAO1 grown in presence and absence of sub-MICs of seed extract. Then, 2 mL of reporter* E. coli* MG4 (pKDT17) strain and 0.5 mL of the ethyl acetate extracted supernatant were incubated at 30°C in a water bath for 5 h with rotation at 100 r.p.m. After centrifugation (3200 g for 15 min) of the reporter cell cultures, cell pellet was suspended in an equal volume of Z buffer (Na_2_HPO_4_·7H_2_O, 0.06 M; NaH_2_PO_4_·H_2_O, 0.04 M; KCl, 0.01 M; MgSO_4_·7H_2_O, 0.001 M; *β*-mercaptoethanol, 0.05 M; pH 7.0). To 1 mL of cell suspension 1 mL of Z buffer, 200 *μ*L of chloroform, and 100 *μ*L of 0.1% sodium dodecyl sulphate were added to lyse cells, and 0.4 mL of o-nitrophenol-*β*-D-galactopyranoside [4 mg/mL in phosphate buffered saline (PBS)] was also added. Reaction was stopped after the development of yellow colour by the addition of 1 mL of 1 M Na_2_CO_3_. OD of the reaction samples was measured at 420 and 550 nm. Units of *β*-galactosidase were calculated as 1000 × OD_420 _nm-(1.75 × OD_550 nm_)/time × volume × OD_600 nm_.

### 2.8. Determination of Effect of Extract on* Caenorhabditis elegans* Survival

The method described by Musthafa et al. [22] was adopted to study the* in vivo* efficiency of* T. foenum-graceum* (seed) extract in* C. elegans* Nematode infection model. Briefly, the young adult Nematodes were infected with PAO1 in the 24-well microtiter plate and incubated at 25°C for 12 h. After incubation,* C. elegans* from the wells was washed thrice with M9 (KH_2_PO_4_ 3 g, Na_2_HPO_4_ 6 g, NaCl 5 g, 1 M MgSO_4_ 1 mL, and distilled water 1000 mL) buffer to remove surface-bound bacteria. Around 10 infected worms were transferred to the wells of microtiter plate containing 10% LB broth in M9 buffer and incubated at 25°C with or without extract.

### 2.9. GC-MS Analysis of Plant Extracts

The composition of the extract was analysed by Perkin Elmer GC Autosystem XL and Turbomass with EI source using PE-Wax column (30 m × 0.25 mm i.d., film thickness 0.25 mm); carrier gas was helium with column head pressure 7 psi connected to data station. The linear temperature program of 60°C to 200°C was set at a rate of 4°C/min^−1^ with hold time at 200°C for 21 minutes. The components were identified by comparing their retention times to those of authentic samples, as well as by comparing their mass spectra with those of Wiley 8 and NIST 05 Libraries. Quantitative data were obtained by the peak normalization technique using integrated FID response.

### 2.10. HPTLC Analysis

Stock standard solutions containing 100 *μ*g/mL of caffeine in methanol were freshly prepared. A sample solution was prepared by dissolving 50 mg of* T. foenum-graceum* (seed) extract in methanol and volume was adjusted to obtain a concentration of 10 mg/mL; the spots were applied on precoated TLC plates with the help of HPTLC applicator Linomat V (CAMAG). The stationary phase used was precoated silica gel 60F_254_ plates (20 cm × 20 cm) from E-Merck and the mobile phase composition was optimized 2-Propanol : Ethyl acetate (4 : 6). The densitometric analysis was performed on CAMAG TLC scanner at 254 nm.

### 2.11. Statistical Analysis

All experiments were performed in triplicate and the data obtained from the experiments were presented as mean values and the differences between control and test were analyzed using Student's *t* test.

## 3. Results

The methanol extract of* T. foenum-graceum *exhibited MIC values of 1200, 2400, and 1200 *μ*g/mL against PAO1, PAF79, and WAF38, respectively.

The extract was evaluated against QS regulated virulence factors in* P. aeruginosa* PAO1 and clinical strain PAF79. At sub-MICs (125, 250, 500, and 1000 *μ*g/mL), the extract showed a concentration dependent effect on virulence factors of* P. aeruginosa* PAO1 (Table 2). Statistically significant reduction (*p* ≤ 0.05) in elastase, total protease, chitinase activity, pyocyanin production, and swarming motility was recorded at the 500 *μ*g/mL concentration. The extract at 1000 *μ*g/mL concentration caused maximum percent decrease in virulence factors such as elastase (61.3%), protease (59.7%), chitinase (47.5%), pyocyanin (55.7%), and swarming motility (59.7%) in* P. aeruginosa* PAO1 over the untreated control (Table 2). However, exopolysaccharide (EPS) produced by the extract treated cultures of PAO1 exhibited a maximum decrease of 46.5% at 1000 *μ*g/mL concentration. In the PAF79 strain, maximum inhibition in the activity of elastase (67.6%), protease (55%), chitinase (87%), pyocyanin production (82.1%), and swarming motility (62.5%) was observed at 1000 *μ*g/mL concentration of extract. EPS production was reduced significantly at all tested concentrations and a maximum decrease of 77.5% was observed at the highest tested concentration of extract (Table 3). The* lasI rhlI* mutant (PAO MW1) grown in the absence of acylated HSL was included as a negative control.

**Figure 1:**
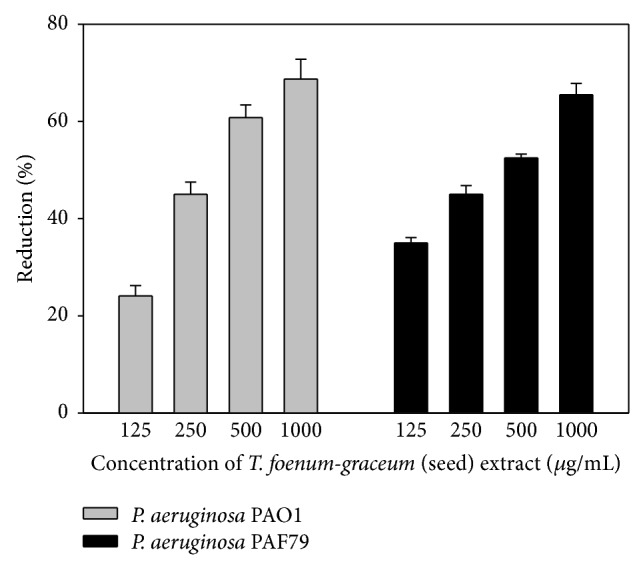
Effect of* T. foenum-graceum* (seed) extract on biofilm formation in* P. aeruginosa* PAO1 and* P. aeruginosa* PAF79 at respective sub-MICs.

**Figure 2:**
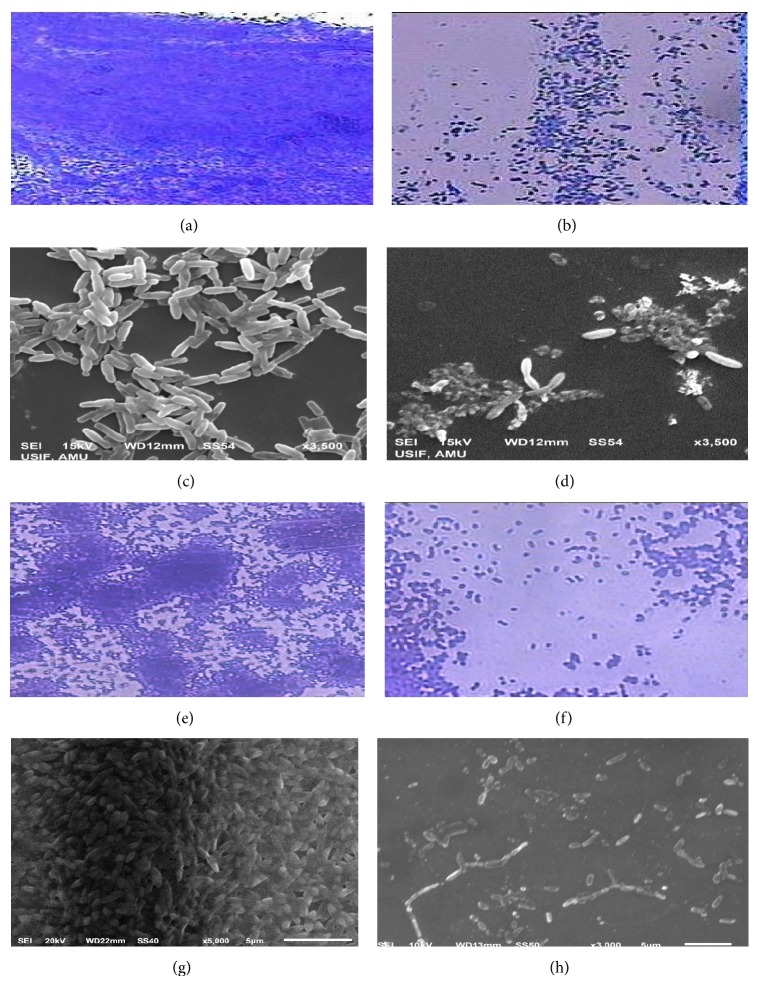
Light and scanning electron microscopic images of* P. aeruginosa* PAO1 and* A. hydrophila* WAF38 biofilm in the presence and absence of sub-MICs of* T. foenum-graceum* (seed) extract: (a, c) untreated control PAO1, (b, d) treated with 1000 *μ*g/mL concentration of* T. foenum-graceum* extract, (e, g) untreated control WAF38, and (f, h) treated with 800 *µ*g/mL concentration of* T. foenum-graceum* extract.

The extract demonstrated a 24.1–68.7% decrease in the biofilm forming ability of PAO1 at sub-MICs tested (125–1000 *μ*g/mL) (Figures 1 and 2). Similarly, significant concentration dependent decrease (*p* ≤ 0.005) in biofilm formation was also observed in PAF79 strain when grown in presence of sub-MICs of the extract. The extract demonstrated a maximum of 65.5% inhibition over untreated control at the highest sub-MIC tested as shown in Figure 1.

The effect of the extract was also assessed against virulence factors of* A. hydrophila* WAF38 at sub-MICs 100–800 *μ*g/mL. Highest tested concentration (800 *μ*g/mL) significantly reduced total protease activity (71.6%), EPS production (46.3%), and biofilm formation (76.9%) (Table 4, Figure 2).

### 3.1. Effect of Methanolic Extract of* Trigonella foenum-graceum* (Seed) on lasB Transcriptional Activity in* E. coli*

**Figure 3:**
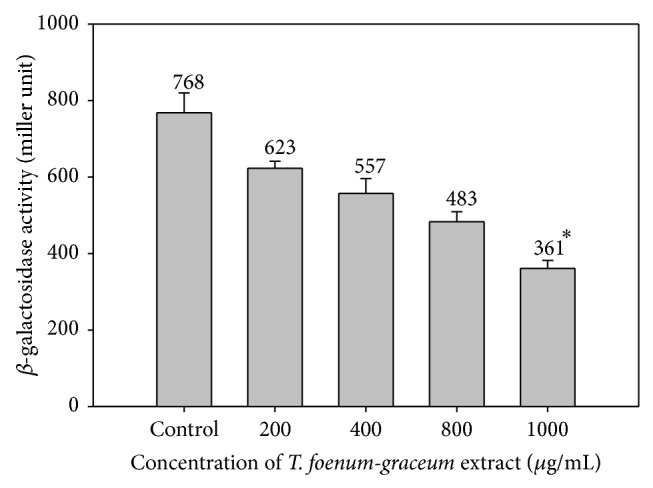
Effect of* T. foenum-graceum* (seed) extract on *β*-galactosidase activity in* E. coli* MG4/pKDT17. All of the data are presented as mean ± SD.  ^∗^Significance at *p* ≤ 0.05 and significance at *p* ≤ 0.005.

Impact of sub-MICs of* T*.* foenum-graceum* (seed) extract on *β*-galactosidase activity of* E. coli* MG4/pKDT17 exhibited a dose dependent decrease. Untreated control produced 768 miller units (MU) whereas 623, 557, 483, and 361 miller units of AHL production were recorded at 200, 400, 800, and 1000 *μ*g/mL concentration (Figure 3). The reduction in the levels of AHL demonstrates that the inhibition of* lasB* promoter activity involves LasR controlled transcription.

### 3.2. Anti-Infective Potential of Seed Extract in* C. elegans* Nematode Model

**Figure 4:**
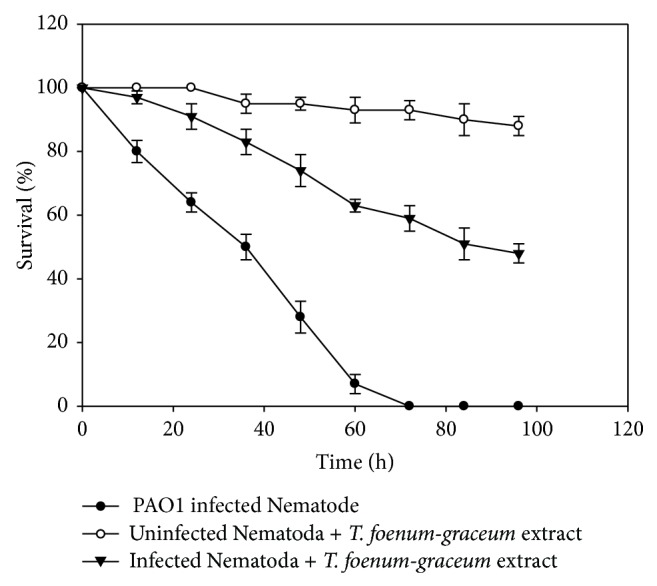
Anti-infection potential of sub-MICs (1000 *μ*g/mL) of* T. foenum-graceum* (seed) extract in increasing survival of preinfected* C. elegans* Nematode model. Means values of triplicate independent experiments and SDs are shown.

In the absence of plant extracts, complete mortality of the PAO1-preinfected* C. elegans* was observed within 72 h, which shows the potent pathogenicity of PAO1 towards the* C. elegans* nematode. However,* C. elegans *preinfected with PAO1 further treated with* T. foenum-graceum* extract (1000 *μ*g/mL) displayed enhanced survival of 48% (Figure 4). However, the extract of* T. foenum-graceum* alone demonstrated no significant effect on the mortality of* C. elegans* at tested concentrations.

### 3.3. GC-MS Analysis

A total of 18 chemical components were identified in methanol seed extract by GC-MS analysis. These numbers may be extended with the help of chemometric techniques. The major compounds identified were 1,3,7-trimethyl-3,7-dihydro-1h-purine-2,6-dione [caffeine] (40.82%) followed by methyl 14-methylpentadecanoate (8.22%), palmitic acid (6.41%), 1,2,3-benzenetriol (6.13%), linoleic acid, methyl ester (5.58%), and capric acid (4.2%). The remaining compounds were present in percentages from 2.01 to 0.1 as depicted in Table 5.

### 3.4. HPTLC Analysis

**Figure 5:**
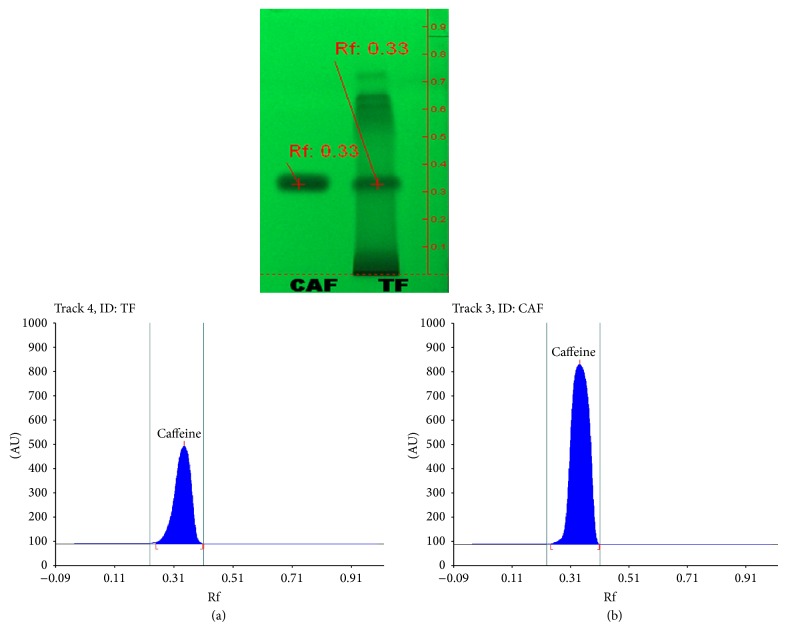
HPTLC chromatogram of* T. foenum-graceum* (seed) extract: (a) methanol extract and (b) pure caffeine.

The mobile phase resolved caffeine efficiently from other components of* T. foenum-graceum *(seed) extract. The Rf of caffeine was found to be 0.33 as shown in Figure 5. The findings of the HPTLC analysis clearly indicate the presence of caffeine in the extract.

### 3.5. Evaluation of QS Inhibitory Activity of Caffeine

**Figure 6:**
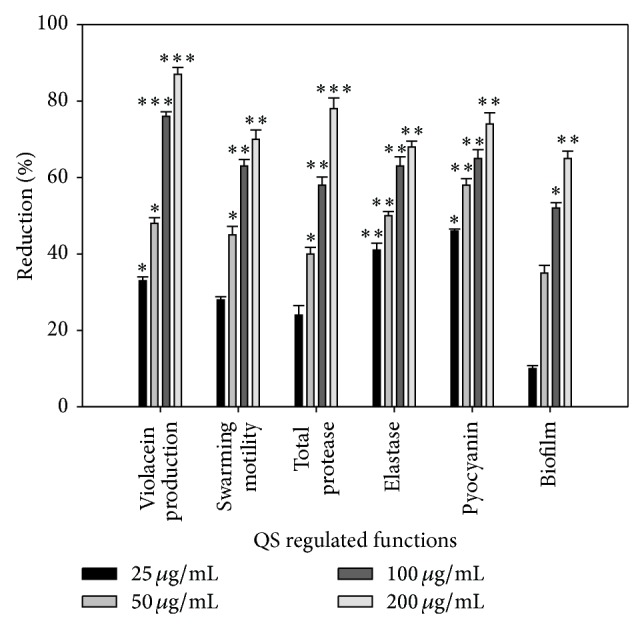
Effect of caffeine on quorum sensing regulated functions and biofilm. All of the data are presented as mean ± SD.  ^∗^Significance at *p* ≤ 0.05,  ^∗∗^significance at *p* ≤ 0.005, and  ^∗∗∗^significance at *p* ≤ 0.001.

Caffeine (1,3,7-trimethyl-3,7-dihydro-1h-purine-2,6-dione) was tested for its possible role in contributing to the anti-QS activity of the plant extract against* Chromobacterium violaceum* CVO26 and* P. aeruginosa* PAO1. Caffeine, at a maximum concentration of 200 *μ*g/mL, showed 87% violacein inhibition in CVO26 (Figure 6). Further, at the same concentration, the compound reduced the biofilm formation (64%), swarming motility (70%), total protease (78%), LasB elastase (68%), and pyocyanin (74%) in* P. aeruginosa* PAO1 (Figure 6). Moreover, caffeine showed no growth inhibitory effect on the test bacterial pathogen.

## 4. Discussion

*P. aeruginosa* proteases and LasB are believed to play a major role in pathogenesis via host tissue degradation [23]. Sub-MICs of* T. foenum-graceum* extract resulted in significant decrease in elastase and protease activities of PAO1 and PAF79. Similar results with other plant extracts were also reported by Adonizio et al. [24] and Packiavathy et al. [25]. It is likely that the extract downregulated LasB activity.

Pyocyanin is another important virulence factor produced under QS regulation. Therefore, the effect of the extracts in reducing the pyocyanin production was assessed and a significant decrease was recorded. By comparison,* Centella asiatica* extract inhibited 80% pyocyanin production at 400 *μ*g/mL [26]. Extracts of some edible plants and fruits also showed similar pyocyanin reduction in PAO1 at sub-MICs [27, 28]. Chitinase plays a major role in the pathogenesis of* P. aeruginosa* in cystic fibrosis patients [29]. Chitinase production by* P. aeruginosa* strains (PAO1 and PAF79) was reduced considerably in a concentration dependent manner under the influence of sub-MICs of* T. foenum-graceum*. The findings are in agreement with our results obtained with sub-MICs of clove oil [20].

It has been well reported that AHL-dependent QS plays a major role in the formation of a biofilm with a complex wild type architecture in many bacteria. In the present study, the extract of* T. foenum-graceum* inhibited the biofilm biomass considerably (*p* ≤ 0.005) in a dose dependent manner without affecting the bacterial growth in both strains of* P. aeruginosa*. Light and scanning electron microscopic images (Figure 2) displayed disintegrated architecture and reduced the number of microcolonies during the biofilm formation of test bacterial pathogens. Therefore, it is envisaged that treatment of bacterial pathogens with the extracts resulted in the formation of weak biofilms possibly by reducing the surface adhesion and subsequent microcolony formation. The results obtained in our study are similar to those reported with the polyphenolic extract of* Rosa rugosa* [30] and standardized extract* of Sclerocarya birrea* [31]. Factors closely associated with biofilm formation of* Pseudomonas aeruginosa *like swarming motility and EPS production were also evaluated in this study. Since, EPS production and swarming motility are under the control of QS, interference with QS would result in the reduced production of EPS and swarming motility. In the present study, the total amount of EPS produced and motility was reduced when bacterial pathogens (PAO1 and PAF79) were treated with sub-MICs of* T. foenum-graceum* extract. Our results find support from the reports on* Capparis spinosa* [32],* Cuminum cyminum* [25] that demonstrated comparable inhibition of swarming motility and EPS production in* P. aeruginosa*. Therefore, it is envisaged that plant extract significantly (*p* ≤ 0.05) reduces EPS and swarming motility which will possibly weaken biofilm formation.

A number of secreted virulence factors are responsible for host tissue destruction during initiation of the infectious process by* Aeromonas* sp. Virulence factors like production of exoproteases, EPS production, and the formation of biofilm are known to be regulated by* ahyRI *QS system [33]. Sub-MICs of the extract demonstrated dose dependent significant reduction in the total protease (*p* ≤ 0.005) and EPS production (*p* ≤ 0.05) in* Aeromonas hydrophila *WAF38. Biofilm formation was also inhibited in a concentration dependent manner by sub-MICs of* T. foenum-graceum* extract. Significant inhibition of biofilm was recorded at all the tested concentrations. The data obtained in our study indicates that the oil is potentially acting on the ahyRI system impairing the production of C4-HSL. Similar significant reduction of total protease, EPS, and biofilm formation of* A. hydrophila* by 0.4% (v/v) of clove oil has been reported by Husain et al. [20].

The addition of extract of* T. foenum-graceum* decreased *β*-galactosidase luminescence in* E. coli* MG4/pKDT17 of this range 18.8–52.9% (*p* ≤ 0.05) at the tested sub-MICs. The results of the assay demonstrate that the reduced production of AHL under the effect of sub-MICs of plant extract inhibits* las*-controlled transcription. The findings of the assay are in agreement with the above observations as reduced *β*-galactosidase activity is indicative of reduced AHL levels and therefore reduced expression of* lasB *gene. Similar observations with other plant extracts were reported by Adonizio et al. [24] in south Florida plants, Singh et al. [34] in* Lagerstroemia speciosa *fruit extract, and Zhou et al. [15] in eugenol.

Further, the protective role of plant extract was assessed in* C. elegans*. Methanol extract of* T. foenum-graceum *displayed an enhanced survival of 48% suggesting that the addition of the extract is affecting the production of cyanide either through* hcn *directly or indirectly via the QS genes. Aqueous extracts of three south Florida plants:* Conocarpus erectus*,* Callistemon viminalis,* and* Bucida buceras* prevented mortality via gut infection in almost 60% of the worms and reduced death from toxins by 50–90% [35].

GC-MS and HPTLC analysis of extract showed the presence of caffeine as one of the major compounds. Studies with caffeine showed significant reduction in AHL regulated production of violacein in CVO26 and QS controlled virulence factors and biofilm formation in* P. aeruginosa* PAO1. Our observations find support from the results demonstrated by Norizan et al. [36]. They showed that caffeine inhibited swarming and AHL production of* P*.* aeruginosa* PA01 and confirmed that caffeine did not degrade AHLs but rather inhibited their production.

Methanol fraction of the* Trigonella foenum-graceum* (seed) extract demonstrated broad spectrum interference of QS regulated functions and inhibited biofilm formation in the gram negative pathogens.* In vitro *attenuation of virulence factors correlated well with the* in vivo *study. Further, caffeine and caffeine related compounds need to be investigated for their possible mechanism of QS interference.

## Figures and Tables

**Table 1:** Bacterial strains used in the study.

Strains	Relevant genotype	Source
*C. violaceum* CVO26	Mini Tn5 mutant of 31532	McLean et al. [13]
*P. aeruginosa* PAO1	Wild type	McLean et al. [13]
*P. aeruginosa* PAO1 MW1	DlasI::Tet DrhlI::Tn501-2 strain PAO1 derivative	Schuster and Greenberg [14]
*E. coli* MG4/pKDT17	*E. coli* DH5*α* harboring plasmid pMG4/pKDT	Zhou et al. [15]
*P. aeruginosa* PAF79	AHL producing strain	Laboratory strain
*A. hydrophila* WAF38	AHL producing strain	Laboratory strain

**Table 2:** Effect of sub-MICs of methanolic extract of *Trigonella foenum-graceum* (seed) on inhibition of quorum sensing regulated virulence factors in *P. aeruginosa *PAO1.

Extract concentration (*μ*g/mL)	Elastase activity^a^	Total protease^b^	Pyocyanin production^c^	Chitinase activity^d^	EPS production^e^	Swarming motility^f^
PAO1-MW1	0.024 ± 0.005	0.061 ± 0.009	0.26 ± 0.012	0.021 ± 0.006	0.118 ± 0.01	9 ± 0.4

Control	0.181 ± 0.044	1.110 ± 0.027	5.2 ± 0.6	0.120 ± 0.009	1.075 ± 0.015	72 ± 1.5

125	0.138 ± 0.040 (23.7)	0.770 ± 0.024 (30.6)	4.3 ± 0.72 (17.3)	0.094 ± 0.003 (21.6)	1.039 ± 0.026 (03.3)	57 ± 3.2 (20.8)

250	0.124 ± 0.037 (31.4)	0.715 ± 0.019 (35.5)	4.0 ± 0.35 (23.0)	0.080 ± 0.013 (33.3)	0.916 ± 0.023 (14.7)	42 ± 1.8 (41.6)^*^

500	0.086 ± 0.020 (52.4)^**^	0.480 ± 0.010 (56.7)^*^	2.8 ± 0.39 (46.1)^*^	0.069 ± 0.013 (42.5)^*^	0.825 ± 0.023 (23.2)	38 ± 1.1 (47.2)^*^

1000	0.070 ± 0.025 (61.3)^**^	0.447 ± 0.008 (59.7)^*^	2.3 ± 0.11 (55.6)^*^	0.063 ± 0.008 (47.5)^*^	0.575 ± 0.016 (46.5)^*^	29 ± 2.0 (59.7)^**^

PAO1-MW1 grown in the absence of acylated HSL was included as a negative control.

^a^Elastase activity is expressed as the absorbance at OD_495_.

^b^Total protease activity is expressed as the absorbance at OD_600_.

^c^Pyocyanin concentrations were expressed as micrograms of pyocyanin produced per microgram of total protein.

^d^Chitinase activity is expressed as the absorbance at OD_570_.

^e^EPS production is expressed as absorbance at OD_480_.

^f^Swarming motility is expressed as diameter of swarm in mm.

The data represents mean values of three independent experiments. ^*^Significance at *p* ≤ 0.05, ^**^significance at *p* ≤ 0.005.

Values in the parentheses indicate percent reduction over control.

**Table 3:** Effect of sub-MICs of methanolic extract of *Trigonella foenum-graceum *(seed) on inhibition of quorum sensing regulated virulence factors in *P. aeruginosa *PAF-79.

Extract concentration (*μ*g/mL)	Elastase activity^a^	Total protease^b^	Pyocyanin production^c^	Chitinase activity^d^	EPS production^e^	Swarming motility^f^
PAO1-MW1	0.029 ± 0.008	0.057 ± 0.004	0.29 ± 0.01	0.020 ± 0.005	0.121 ± 0.014	8 ± 0.7

Control	0.167 ± 0.025	1.039 ± 0.041	3.8 ± 0.25	0.139 ± 0.005	0.886 ± 0.036	48 ± 1.5

125	0.105 ± 0.022 (37.1)	0.564 ± 0.024 (45.7)^*^	2.6 ± 0.42 (31.5)^*^	0.094 ± 0.016 (32.3)	0.414 ± 0.025 (53.2)^*^	39 ± 2 (18.7)

250	0.085 ± 0.021 (49.1)^*^	0.547 ± 0.033 (47.3)^*^	1.2 ± 0.27 (68.4)^**^	0.065 ± 0.023 (53.3)^*^	0.357 ± 0.019 (59.7)^**^	32 ± 2 (33.3)^*^

500	0.057 ± 0.016 (65.8)^*^	0.499 ± 0.003 (51.9)^*^	0.94 ± 0.016 (75.2)^**^	0.038 ± 0.006 (72.6)^**^	0.309 ± 0.031 (65.1)^**^	23 ± 0.5 (52)^*^

1000	0.054 ± 0.003 (67.6)^*^	0.468 ± 0.010 (55)^*^	0.68 ± 0.028 (82.1)^***^	0.018 ± 0.004 (87)^***^	0.199 ± 0.014 (77.5)^**^	18 ± 3 (62.5)^*^

PAO1-MW1 grown in the absence of acylated HSL was included as a negative control.

^a^Elastase activity is expressed as the absorbance at OD_495_.

^b^Total protease activity is expressed as the absorbance at OD_600_.

^c^Pyocyanin concentrations were expressed as micrograms of pyocyanin produced per microgram of total protein.

^d^Chitinase activity is expressed as the absorbance at OD_570_.

^e^EPS production is expressed as absorbance at OD_480_.

^f^Swarming motility is expressed as diameter of swarm in mm.

The data represents mean values of three independent experiments. ^*^Significance at *p* ≤ 0.05, ^**^significance at *p* ≤ 0.005, and ^***^significance at *p* ≤ 0.001.

Values in the parentheses indicate percent reduction over control.

**Table 4:** Effect of sub-MICs of methanolic extract of *Trigonella foenum-graceum *(seed) on inhibition of quorum sensing regulated virulence factorsin *Aeromonas hydrophila* WAF-38.

Concentration (*μ*g/mL)	Total protease^a^	EPS production^b^	Biofilm formation^c^
Control	0.589 ± 0.051	0.748 ± 0.021	0.226 ± 0.006

100	0.520 ± 0.025 (11.7)	0.721 ± 0.064 (03.6)	0.126 ± 0.011 (44.2)^*^

200	0.431 ± 0.049 (26.8)	0.642 ± 0.035 (14.1)	0.077 ± 0.018 (65.9)^**^

400	0.321 ± 0.033 (45.5)^*^	0.516 ± 0.013 (31.0)	0.068 ± 0.004 (69.9)^**^

800	0.167 ± 0.018 (71.6)^**^	0.401 ± 0.021 (46.3)^*^	0.052 ± 0.010 (76.9)^**^

^a^Total protease activity is expressed as the absorbance at OD_600_.

^b^EPS production is expressed as absorbance at OD_480_.

^c^Biofilm formation is expressed as OD_470_ after incubation with crystal violet.

The data represents mean values of three independent experiments. ^*^Significance at *p* ≤ 0.05, ^**^significance at *p* ≤ 0.005.

Values in the parentheses indicate percent reduction over control.

**Table 5:** Components of *Trigonella foenum-graceum* (seed) extractas identified by GC-MS analysis.

Peak number	Components	Retention time	Area (%)
1	1,2,3-Benzenetriol	8.76	6.13
2	Capric acid	11.42	4.20
3	1,3,7-Trimethyl-3,7-dihydro-1h-purine-2, and 6-dione (Caffeine)	13.85	40.82
4	Methyl 14-methylpentadecanoate	14.29	8.22
5	Palmitic acid	14.70	6.41
6	Linoleic acid, methyl ester	15.93	5.58
7	9,12,15-Octadecatrienoic acid, methyl ester	16.00	9.17
8	Phytol	16.11	1.45
9	Methyl stearate	16.20	1.75
10	10,12-Hexadecadien-1-ol	16.34	2.56
11	cis-11-Eicosenoic acid, methyl ester	17.75	0.43
12	Palmidrol	17.89	0.10
13	Methyl 18-methylnonadecanoate	17.95	0.12
14	Hexacosane	18.54	0.11
15	1-Monopalmitin trimethylsilyl ether	19.37	0.13
16	Methyl 18-methylnonadecanoate	22.87	0.40
17	2-Hydroxy-3-(palmitoyloxy)propyl (9E)-9-octadecenoate	28.14	1.48
18	Stigmasterol	29.46	2.01
19	Stigmast-8(14)-en-3.beta.-ol	29.98	0.48
